# High PHLPP1 expression levels predicts longer time of acquired resistance to EGFR tyrosine kinase inhibitors in patients with lung adenocarcinoma

**DOI:** 10.18632/oncotarget.19777

**Published:** 2017-08-01

**Authors:** Youyou Xie, Dongqing Lv, Wei Wang, Minhua Ye, Xiaofeng Chen, Haihua Yang

**Affiliations:** ^1^ Laboratory of Cellular and Molecular Radiation Oncology, Affiliated Taizhou Hospital of Wenzhou Medical University, Taizhou, 317000, Zhejiang Province, China; ^2^ Department of Radiation Oncology, Affiliated Taizhou Hospital of Wenzhou Medical University, Taizhou, 317000, Zhejiang Province, China; ^3^ Department of Pulmonary Medicine, Affiliated Taizhou Hospital of Wenzhou Medical University, Taizhou, 317000, Zhejiang Province, China; ^4^ Department of Thoracic Surgery, Affiliated Taizhou Hospital of Wenzhou Medical University, Taizhou, 317000, Zhejiang Province, China; ^5^ Enze Medical Research Center, Affiliated Taizhou Hospital of Wenzhou Medical University, Taizhou, 317000, Zhejiang Province, China

**Keywords:** lung adenocarcinoma, PH domain leucine-rich-repeats protein phosphatase, epidermal growth factor receptor tyrosine kinase inhibitor, acquired resistance

## Abstract

**Background:**

In spite of an initial good response to epidermal growth factor receptor tyrosine kinase inhibitors (EGFR TKIs) in lung adenocarcinoma patients, resistance to treatment eventually occurs. Epidermal growth factor receptor (EGFR) activation stimulates Ras/Raf/Erk/MAPK and influences PI3K/Akt pathways, respectively. PHLPP negatively regulates PI3K/Akt and the RAF/RAS/ERK signaling pathways. Our study aimed to investigate the association between PH domain leucine-rich-repeats protein phosphatase (PHLPP) expression levels and the acquired resistance to EGFR TKIs in lung adenocarcinoma.

**Results:**

High expression levels of PHLPP1 and PHLPP2 were detected in 69.3% and 61.3%, respectively, of patients with lung adenocarcinoma. Patients with high expression levels of PHLPP1 showed significantly longer median progression-free survival and overall survival than those with low expression levels of PHLPP1 (29 months versus 11 months, and 36 months versus 19 months respectively) (*p* = 0.0050 and *p* = 0.0052). PHLPP1, but not PHLPP2, protein expression levels was negatively correlated with p-Akt (473) and p-Erk1/2. The PHLPP1 expression levels were correlated with Progression-free survival and overall survival (*p* = 0.001 and *p* = 0.000).

**Materials and Methods:**

We recruited 75 patients with advanced lung adenocarcinoma receiving EGFR TKIs treatment. The expression levels of PHLPP1, PHLPP2, p-AKT(S473) and p-ERK1/2 were assessed using tissue immunostaining. The association of PHLPP expression levels with clinicopathological parameters and disease prognosis was analyzed.

**Conclusions:**

This study suggests that high expression levels of PHLPP1 predict a better survival from target therapy and a longer time of acquired resistance to EGFR TKIs in patients with lung adenocarcinoma.

## INTRODUCTION

Lung cancer is the most common form of cancers with a high mortality [1]. Non-small-cell lung cancer (NSCLC) accounts for approximately 80% of lung cancers, in which adenocarcinoma accounts for about 38% [2]. Unfortunately, most NSCLC patients are diagnosed at advanced stage with a poor prognosis [3]. Epidermal growth factor receptor (EGFR) is overexpressed in most NSCLC and represents a major therapeutic target. Recently, the discovery of EGFR-tyrosine kinase inhibitors (TKIs) such as gefitinib and erlotinib led to great success in clinical treatment [4, 5]. EGFR tyrosine kinase inhibitor (TKI) treatment effectively improves survival of NSCLC patients with an EGFR mutation [6, 7].

Although EGFR-TKI treatment exhibited initial beneficial effects in most patients with NSCLC, these patients eventually developed resistance to EGFR-TKIs, with disease progression within approximately a year [8, 9]. Several mechanisms have been proposed for this resistance, including a secondary mutation in EGFR (such as T790M), amplification of the MET receptor tyrosine kinase, and other receptor tyrosine kinase overexpression.

PH domain leucine-rich-repeats protein phosphatase (PHLPP) belongs to a novel family of Ser/Thr protein phosphatases. There are two isoforms of PHLPP in its phosphatase family, PHLPP1 and PHLPP2, which share ~50% identity at the amino acid level. PHLPP negatively regulates PI3K/Akt and RAF/RAS/ERK signaling pathways [10, 11]. PI3K/Akt pathway is important in inhibiting apoptosis [12]. The RAF/RAS/ERK pathway contributes to proliferation differentiation and motility. EGFR activation triggers the Ras/Raf/Erk/MAPK, and stimulates the PI3K/Akt, pathways. Therefore, activation of proteins in PI3K/Akt pathway could also potentially lead to TKI resistance. Conversely, PHLPP as a negative regulator of PI3K/Akt and RAF/RAS/ERK pathways might slow down the process of resistance.

The aim of our study was to investigate the association between PHLPP expression and its effect on target-based therapies and acquired resistance to EGFR-TKI in lung adenocarcinoma.

## RESULTS

**Figure 1:**
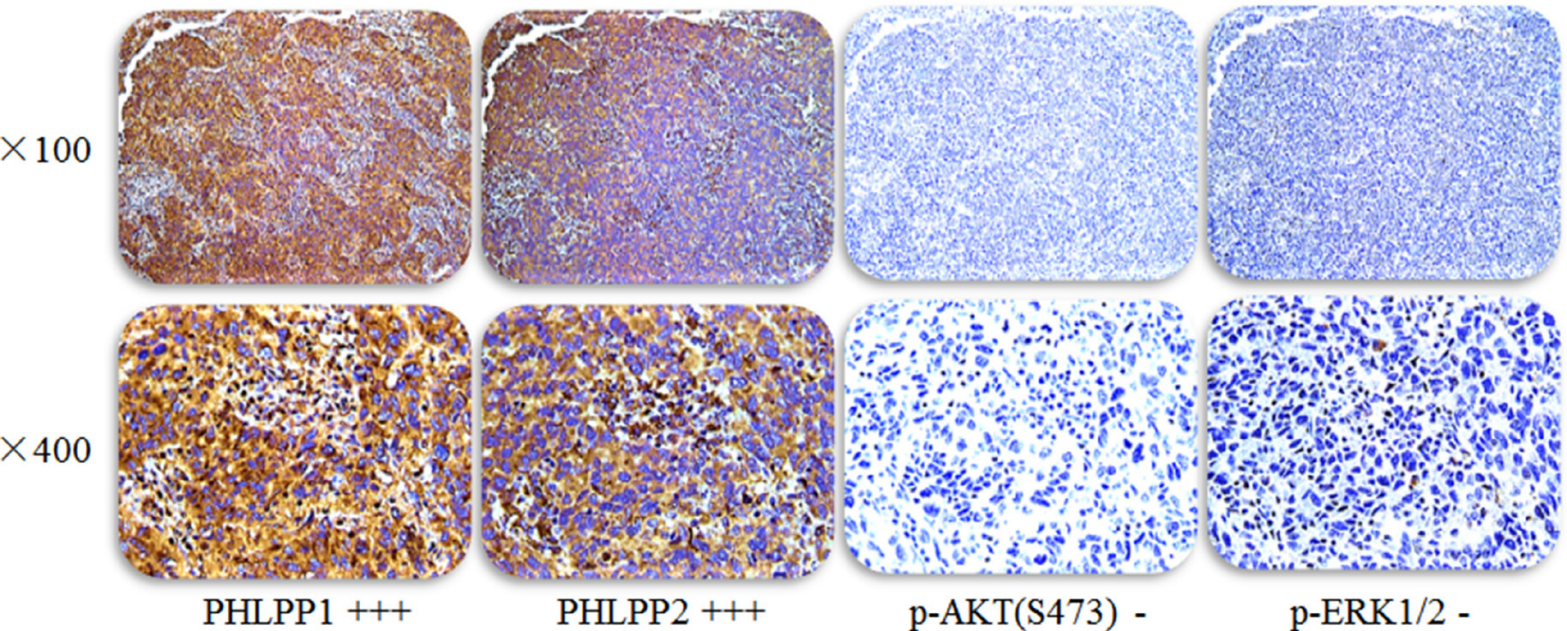
Representative images of PHLPP1 and PHLPP2 and p-AKT(S473) and p-ERK1/2 expression in the same lung adenocarcinoma patient with EGFR TKI treatment effectively Staining in tissues was evaluated by three pathologists who were blinded to any clinical details related to the patients. The entire section was assessed at low (100×) (upper panels) and high (400×) power (below panels) magnification.

Among the 75 patients with histologically proven lung adenocarcinoma, 33 (44%) were male and 42 (56%) were female. The median age was 60 years. All patients were treated with gefitinib or erlotinib and showed a complete response (CR), partial response (PR), or Stable disease (SD). The expression levels of PHLPP1 and PHLPP2 and p-Akt (S473) and p-Erk1/2 (Figure 1) in lung adenocarcinoma were assessed by immunohistochemistry. High (2–3 point) and low (0–1 point) expression levels of PHLPP1 were noted in 52 (69%) and 23 (30.67%) patients, respectively. Correlations between PHLPP1 abundance and clinicopathological characteristics were summarized in Table 1. High (2–3 point) and low (2–3 point) expression levels of PHLPP2 (2–3 point) were found in 46 (61.3%) and 29 (38.7%) patients, respectively. Correlations between PHLPP2 abundance and clinicopathological characteristics were summarized in Supplementary Table 1.

**Table 1:** PHLPP 1 and baseline characteristics of patients

	No. of case	PHLPP 1 expression		*P* value
		High expression	Low expression	
Age (Median ***=*** 60 yeas)				
≤ 60 years	38	28	10	0.408
**>** 60 years	37	24	13	
Gender				
Male	33	20	13	0.146
Female	42	32	10	
Smoking Status				
Ever-smoker	19	10	9	0.068
Never-smoker	56	42	14	
EGFR-TKI				
Erlotinib	38	23	15	0.094
Gefitinib	37	29	8	
T				
1	21	15	6	0.561
2	39	27	12	
3	12	7	5	
4	3	3	0	
N				
0	37	30	7	0.108
1	20	13	7	
2	13	7	6	
3	5	2	3	
p-Akt (S473)				
Positive	14	4	10	0.001
Negative	61	48	13	
p-ERK 1/2				
Positive	24	6	18	0.000
Negative	51	46	5	
EGFR				
Mutation	42	28	14	0.572
Unknown	33	24	9	

**Figure 2:**
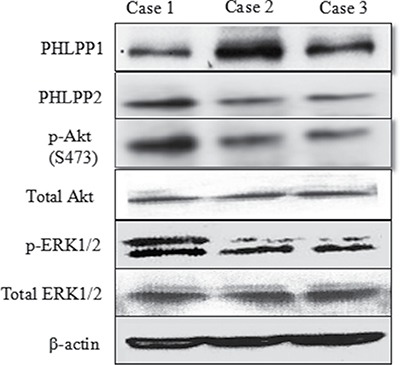
Representative images of Western blot of PHLPP and p-Akt and p-ERK in fresh tissue of three typical patients Choose three typical cases include: case 1 without progress is less than half a year, case 2 with no progress is more than 2 years, and case 3 with no progress in 1 to 1.5 years. A negative correlation was noted between PHLPP protein expression with p-Akt and p-ERK in fresh tissue of patients.

In Figure 2, Case 1, a 65-year-old smoker receiving gefitinib therapy was progressive free for 5 months. The patient did not join the medical donation project and died. Case 2 was a 67-year-old nonsmoking female with both gefitinib therapy who received free drugs from the medical donation project. She is still living and has been progression free for 28 months. Case 3 was a 57-year-old non-smoking female with Tarceva therapy who also received the drug from the medical donation project. She was progression free for 18 months, but died later. PHLPP protein expression was negatively correlated with p-Akt(S473) and p-ERK1/2.

**Figure 3:**
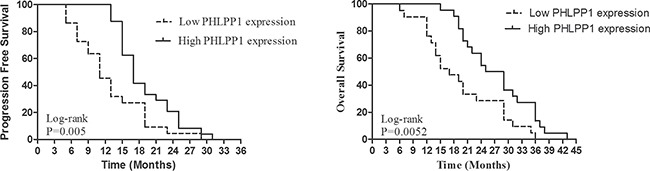
Kaplan-Meier PFS and OS curves of patients with high and low expression of PHLPP1 Patients with high expression of PHLPP1 showed significantly longer PFS and OS than those with low expression of PHLPP1 (14 months versus 7 months, 31 months versus 25 months, respectively).

The PFS and OS were independently correlated with PHLPP1 expression levels. The survival curves are shown in Figure 3. PFS and OS were significantly better in patients with high expression levels of PHLPP1 than those with low expression levels of PHLPP1 (*p* = 0.0050 and 0.0052). Patients with high expression levels of PHLPP1 showed significantly longer OS and higher PFS than those with low expression levels of PHLPP1 (36 months versus 19 months, 29 months versus 11 months respectively). In univariate and multivariate analyses, PHLPP1 was independently prognostic of PFS (Table 2 and Table 3) and OS (Supplementary Table 2 and Supplementary Table 3 ). PHLPP2 has no impact on either PFS or OS (Table 2 and Supplementary Table 2 and Supplementary Figure 1).

**Table 2:** Univariate analysis of PFS in patients with lung adenocarcinoma treated with EGFR-TKI

Variables		HR	95% CI	*p* value
Age	≤ 60 years vs. > 60years (Ref.)	0.858	0.479–1.534	0.605
Gender	Female vs. Male (Ref)	0.617	0.346–1.101	0.103
Smoking Status	Never-smoker vs. Ever-smoker (Ref)	0.448	0.244–0.824	0.010
EGFR-TKI	Erlotinib vs. Gefitinib (Ref)	0.763	0.422–1.381	0.372
T	1 and 2 vs. 3 and 4 (Ref)	1.508	0.764–2.975	0.236
N	0 and 1 vs. 2 and 3 (Ref)	2.421	1.380–4.248	0.002
PHLPP1 expression	High expression vs. Low expression (Ref)	0.210	0.116–1.381	0.000
PHLPP2 expression	High expression vs. Low expression (Ref)	1.032	0.566–1.880	0.919
pAKT (S473) expression	High expression vs. Low expression (Ref)	2.542	1.333–0.380	0.005
pERK 1/2 expression	High expression vs. Low expression (Ref)	2.444	1.359–4.393	0.003

**Table 3:** Multivariate analysis of PFS in patients with lung adenocarcinoma treated with EGFR-TKI

Variables		HR	95% CI	*p* value
Smoking Status	Never-smoker vs. Ever-smoker (Ref)	0.488	0.236–1.007	0.052
N	0 and 1 vs. 2 and 3 (Ref)	1.488	0.825–2.683	0.187
PHLPP1 expression	High expression vs. Low expression (Ref)	0.232	0.123–0.438	0.000
pAKT (S473) expression	High expression vs. Low expression (Ref)	1.439	0.634–3.266	0.384
pERK 1/2 expression	High expression vs. Low expression (Ref)	0.666	0.339–1.310	0.239

## DISCUSSION

Specific EGFR-TKIs, such as gefitinib and erlotinib, are the first line medication for advanced NSCLC. However, published studies have revealed that the clinical response to EGFR tyrosine kinase inhibitors varies in patient cohorts. Treatment with gefitinib was associated with significantly greater survival for patients with refractory advanced NSCLC in non-smokers or Asians [14]. Additionally, gefitinib provided survival benefit in a subgroup of NSCLC patients with adenocarcinoma histology and any degree of skin rash following therapy [15]. Probably, EGFR somatic mutation is the most effective molecular predictor for EGFR-TKIs’ responsiveness and efficacy. The EGFR mutations are present in four exons (18–21 exons) of the tyrosine kinase domain of EGFR. EGFR mutation frequency vary in patients’ ethics, smoking status and histology [16–17]. It has been shown that EGFR-TKI therapeutic efficacy in advanced NSCLC with EGFR mutations can reach 60–80% and PFS can be longer than one year in many large clinical studies [9, 18–20]. However, acquired EGFR-TKI resistance is still inevitable although the precise underlying mechanisms remain unclear [21].

To find effective biological factors associated with EGFR-TKIs responsiveness and to provide the most direct and valuable guidance for clinicians to make decisions on EGFR-TKIs therapy has been a big challenge. It has been reported that 50–60% of cases with the resistance to EGFR-TKIs were due to a second T790M mutation [22] and that 5–22% resulted from gene amplification of mesenchymal-epithelial transition (MET) [23]. Other factors such as small-cell lung cancer transformation, hepatocyte growth factor (HGF) overexpression, Gas6-Axl activation, Loss of phosphatase and pensin homolog (PTEN), epithelial–mesenchymal transition (EMT) and activating mutations in phosphatidylinositol-4,5-bisphosphate 3- kinase (PI3K) have also been reported to associate with acquired EGFR-TKI resistance [24–29].

PHLPP negatively regulates PI3K/Akt [10] and RAF/RAS/ERK in cancer cells [11]. As demonstrated previously, both Akt and ERK contribute to carcinogenesis, chemoresistance [30, 31] and the tolerance of chemoradiotherapy in lung cancer [32]. Several recent studies have provided strong evidence that PHLPP serves as an important tumor suppressor in cancers such as colon cancer, glioblastoma and prostate cancer [33–35]. We previously reported the high expression rate (23.4%) of PHLPP in lung adenocarcinoma. Additionally, PHLPP expression is significantly correlated with differentiation stage and local tumor T-stage in lung adenocarcinoma, whereas low PHLPP expression is associated with poor prognosis in patients with resected lung adenocarcinoma [13]. In the present study, however, the high expression rate of PHLPP1 (2–3 point) reached 69.3%. One possibility is that two studies used different PHLPP antibodies. One was mainly to detect its membrane expression (Abcam), whereas another was to detect its cytoplasm expression (ProteinTech). Another possibility is that all patients in the present study were all EGFR TKI treating sensitive, with a better prognosis than patients in our previous study.

We previously found that PHLPP expression significantly correlated with survival time in lung adenocarcinoma patients [13]. The current study directly examined the association between PHLPP1 expression and the efficacy of target therapy in human lung adenocarcinoma. We further demonstrated that the efficacy of target therapy was better in patients with high expression of PHLPP1. Additionally, the time of acquired resistance to EGFR-TKI in NSCLC was much longer in patients with high expression of PHLPP1. Of note, PHLPP1 still inversely correlated with the expression of p-Akt and/or p-ERK in human lung adenocarcinoma tissues.

High expression of PHLPP1 in lung adenocarcinoma highly correlated with longer survival. The molecular mechanism responsible for the correlation between PHLPP1 expression with the outcome of NSCLC patients receiving EGFR-TKIs is unclear. One possible explanation is that both PI3K/Akt and RAF/RAS/ERK pathways are the downstream signaling molecules of EGFR signaling, which is associated with the occurrence and development of lung cancer. Also, it has been reported that PHLPP isozymes set the amplitude of receptor tyrosine kinase (RTK) signaling by serving as regulators of RTK transcription [36]. Therefore, PHLPP as a tumor suppressor shows an even broader role. It may be a potential prognostic marker for screening patients for target therapy.

In conclusion, our study suggests that high levels of PHLPP1 might predict a better survival of target therapy and a longer time before acquired resistance to EGFR-TKI in lung adenocarcinoma patients. PHLPP1 might be a potential prognostic marker to screen patients for target therapy. To the best of our knowledge, this study for the first time demonstrates the relationship between efficacy of EGFR-TKI target therapy and PHLPP1 expression. However, it is a single center study and the number of subjects studied is limited. Therefore, future larger multi-center trials with larger sample sizes may be necessary to generalize the results.

## MATERIALS AND METHODS

### Patients

We recruited 75 patients diagnosed with advanced lung adenocarcinoma and having received treatment with a single agent EGFR-TKI and exhibiting objective therapeutic benefit from 2008 to 2012 at Taizhou Hospital of Zhejiang Province in China. The Eastern Cooperative Oncology Group (ECOG) performance status scores of the recruited patients were from 0 to 2. All patients had adequate organ function as defined as a white blood cell (WBC) counts > 4 × 10^9^/dl (absolute granulocyte > 2 × 10^9^/dl), platelet > 100 × 10^9^/dl, normal liver function tests and serum creatinine level < 1.4 mg/dl, and no other severe comorbid conditions. Patient stage was redetermined according to the TNM Staging System of AJCC (7th version, 2009). All patients had distant metastases (M1). All patients gave informed consent. This study was granted by the Ethics Committee of Taizhou Hospital and tissue specimen acquisition was performed in agreement with institutional guidelines.

### Immunohistochemistry

The presence of PHLPP and p-AKT and p-ERK in lung tissues was assessed by immunohistochemistry (IHC) as we previously reported [13]. Antibody information is: PHLPP1 (1:200, 22789-1-AP, ProteinTech Group, Chicago, USA), PHLPP2 (1:200, 25244-1-AP, ProteinTech Group, Chicago, USA), p-AKT(S473) (1:200, BS4007, Bioworld Technology, MN, USA) and p-ERK 1/2(BS5016, 1:200, Bioworld Technology, MN, USA). Three pathologists, who were blind to all clinical and biological data, performed and analyzed IHC. A four tier system (level 0–3: negative = 0, weakest = 1, moderate = 2, strong = 3) was used to grade the intensity of staining [14]. The group with low PHLPP low expression had 0–1 point, and the PHLPP high expression group was scored as 2–3 point. To confirm the specificity of IHC, two types of negative controls were used, which included substituting rabbit non-immune IgG for primary antibodies, and omitting primary antibodies to rule out potential non specificity of secondary antibodies. Positive staining was confirmed in normal colonic mucosa slide which was used as a positive control.

### Western blotting

Fresh tissue was collected prior to targeted therapy and was snap-frozen in liquid nitrogen and stored at – 80°C. Three typical cases were chosen: case 1 without progress in less than half a year, case 2 with no progress in more than 2 years, and case 3 with no progress in 1 to 1.5 years. Tissues were extracted in a lysis buffer (pH7.4, 50 mM Tris-base, 150 mM NaCl, 1 mM EDTA, 1% Triton X-100). Proteins extracted from the tissues were loaded onto 10% SDSPAGE and electrophoretically transferred to PVDF membranes (Millipore, USA). After blocking with 5% non-fat dry milk for 1 hour, membranes were washed in PBS containing 0.2% Tween-20 and then incubated in the corresponding primary antibodies overnight at 4°C. The membranes were subsequently incubated with Peroxidase-conjugated Affinipure Goat Anti-Rabbit IgG Fc fragment (Dako, USA) for 1 hour at room temperature. Immunoreactivity was visualized using Biomax film (Kodak, USA) for 1–5 min in the dark room after incubation of membranes in an enhanced chemiluminescence reagent (ECL Plus Western Blotting Detection Systems, Amersham Biosciences, USA).

### Patient follow-up

All patients received initial treatment with a single administration of EGFR-TKI and exhibited objective clinical benefit from the treatment. Acquired resistance was defined if patients experienced disease progression during continuous EGFR-TKI treatment. Each patients’ record included age, sex, smoking status, the medicine type (gefitinib or erlotinib), date of diagnosis, date of acquired resistance, and time of follow-up which was every 3 months.

### Statistics

Statistical analyses were performed using SPSS for Windows (version 21.0). Chi-squared test was used for count data. Progression-free survival (PFS) and overall survival (OS) were estimated using the Kaplan-Meier method. PFS was defined from the date of gefitinib or erlotinib initiation until the date of first documented progression. OS was defined from the date of gefitinib or erlotinib initiation until the date of death. *P*-value < 0.05 represents statistical significance.

## SUPPLEMENTARY MATERIALS FIGURE AND TABLES



## Abbreviations

NSCLC: Non-small-cell lung cancer
EGFR: epidermal growth factor receptor
TKIs: tyrosine kinase inhibitors
PHLPP: PH domain leucine-rich-repeats protein phosphatase
MET: mesenchymal-epithelial transition
PTEN: Phosphatase and Tensin Homolog
EMT: epithelial–mesenchymal transition
PI3K: phosphatidylinositol-4,5-bisphosphate 3- kinase
RTK: receptor tyrosine kinase
