# Supplementary material for: High PHLPP1 expression levels predicts longer time of acquired resistance to EGFR tyrosine kinase inhibitors in patients with lung adenocarcinoma
Source: Oncotarget. 2017 Aug 1;8(35):59000–7. doi: 10.18632/oncotarget.19777 (PMC5601709; doi:10.18632/oncotarget.19777)
Supplement: Supplementary file 1 [file oncotarget-08-59000-s001.pdf]

High PHLPP1 expression levels predicts longer time of acquired resistance to EGFR tyrosine kinase inhibitors in patients with lung adenocarcinoma

SUPPLEMENTARY MATERIALS

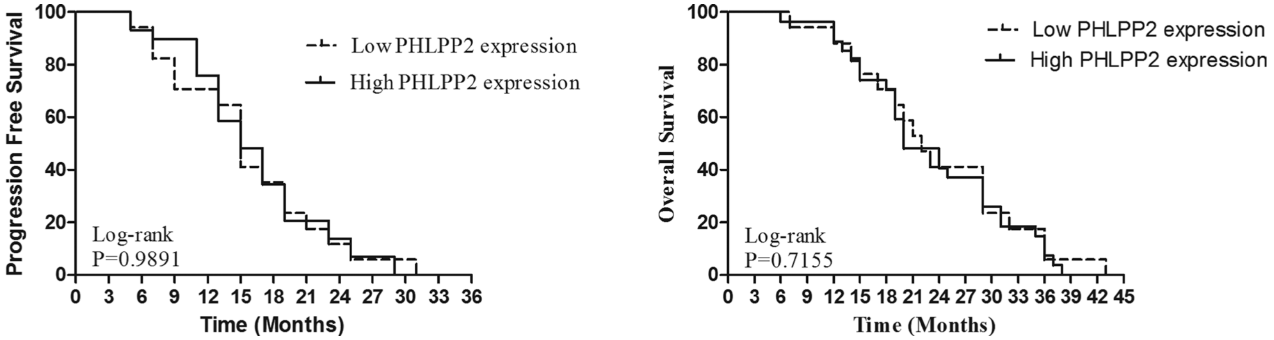

Supplementary Figure 1: Kaplan-Meier PFS and OS curves of patients with high and low expression of PHLPP2. Patients with high expression of PHLPP2 showed significantly longer PFS and OS than those with low expression of PHLPP2 (15 months versus 15 months, 20 months versus 22 months, respectively).

Supplementary Table 1: PHLPP 2 and baseline characteristics of patients

	No. of case	PHLPP2 expression		*P* value
		High expression	Low expression	
Age (Median = 60 yeas)				
≤ 60 years	38	24	14	0.742
> 60 years	37	22	15	
Gender				
Male	33	22	11	0.401
Female	42	24	18	
Smoking Status				
Ever-smoker	19	10	9	0.837
Never-smoker	56	31	25	
EGFR-TKI				
Erlotinib	38	26	12	0.201
Gefitinib	37	20	17	
T				
1	21	14	7	0.693
2	39	23	16	
3	12	8	4	
4	3	1	2	
N				
0	37	26	11	0.415
1	20	11	9	
2	13	7	6	
3	5	2	3	
p-Akt (S473)				
Positive	14	9	5	0.801
Negative	61	37	24	
p-ERK 1/2				
Positive	24	14	10	0.714
Negative	51	32	19	
EGFR				
Mutation	42	25	17	0.717
Unknown	33	21	12	

Supplementary Table 2: Univariate analysis of overall survival in patients with lung adenocarcinoma treated with EGFR-TKI

Variables		HR	95% CI	*p* value
Age	≤ 60 years vs.> 60years (Ref.)	1.012	0.554–1.847	0.970
Gender	Female vs. Male (Ref)	0.570	0.313–1.039	0.067
Smoking Status	Never-smoker vs. Ever-smoker (Ref)	0.530	0.284–0.990	0.047
EGFR-TKI	Erlotinib vs. Gefitinib (Ref)	0.975	0.518–1.835	0.938
T	1 and 2 vs. 3 and 4 (Ref)	1.774	0.852–3.692	0.126
N	0 and 1 vs. 2 and 3 (Ref)	2.095	1.189–3.693	0.011
PHLPP1 expression	High expression vs. Low expression (Ref)	0.284	0.155–0.518	0.000
PHLPP2 expression	High expression vs. Low expression (Ref)	0.933	0.501–1.737	0.827
pAKT (S473) expression	High expression vs. Low expression (Ref)	1.561	0.765–3.185	0.221
pERK 1/2 expression	High expression vs. Low expression (Ref)	0.581	0.301–1.121	0.105

Supplementary Table 3: Multivariate analysis of overall survival in patients with lung adenocarcinoma treated with EGFR-TKI

Variables		HR	95%CI	*p* value
Gender	Female vs. Male (Ref)	0.687	0.274–1.679	0.401
Smoking Status	Never-smoker vs. Ever-smoker (Ref)	0.664	0.246–1.797	0.420
N	0 and 1 vs. 2 and 3 (Ref)	1.135	0.595–2.166	0.701
PHLPP1 expression	High expression vs. Low expression (Ref)	0.298	0.152–0.584	0.000
pAKT (S473) expression	High expression vs. Low expression (Ref)	1.679	0.491–3.001	0.675
pERK 1/2 expression	High expression vs. Low expression (Ref)	0.577	0.283–1.176	0.130
